# A national public health programme on gambling policy development in New Zealand: insights from a process evaluation

**DOI:** 10.1186/s12954-018-0217-y

**Published:** 2018-03-06

**Authors:** Komathi Kolandai-Matchett, Jason Landon, Maria Bellringer, Max Abbott

**Affiliations:** Gambling and Addictions Research Centre, Faculty of Health and Environmental Sciences, Auckland University of Technology, Private Bag 92006, Auckland, 1142 New Zealand

**Keywords:** Workplace, organisational, fundraising, and electronic gaming machine gambling policies, Gambling harm reduction, Problem gambling public health programme

## Abstract

**Background:**

In New Zealand, a public health programme on gambling policy development is part of a national gambling harm reduction and prevention strategy mandated by the *Gambling Act 2003*. Funded by the Ministry of Health, the programme directs *workplace/organisational gambling policies*, *non-gambling fundraising policies*, and local *council policies on electronic gaming machines* (EGMs). We carried out a *process evaluation* of this programme to identify practical information (e.g. advocacy approaches; challenges and ameliorating strategies) that can be used by programme planners and implementers to reinforce programme effectiveness and serve to guide similar policy-focused public health initiatives elsewhere.

**Methods:**

Evaluation criteria, based on the programme’s official *service specifications*, guided our evaluation questions, analysis and reporting. To identify informative aspects of programme delivery, we thematically analysed over 100 six-monthly implementer progress reports (representing 3 years of programme delivery) and transcript of a focus group with public health staff.

**Results:**

Identified output-related themes included purposeful awareness raising to build understanding about gambling harms and the need for harm-reduction policies and stakeholder relationship development. Outcome-related themes included enhanced community awareness about gambling harms, community involvement in policy development, some workplace/organisational policy development, and some influences on council EGM policies. Non-gambling fundraising policy development was not common.

**Conclusions:**

The programme offers an unprecedented gambling harm reduction approach. Although complex (due to its three distinct policy focus areas targeting different sectors) and challenging (due to the extensive time and resources needed to develop relationships and overcome counteractive views), the programme resulted in some policy development. Encouraging workplace/organisational policy development requires increased awareness of costs to employers and society and appreciation of policy value. Although encouraging non-gambling fundraising policies will likely remain challenging, public debate on ethical aspects could stimulate policy consideration. Influencing council EGM policy decisions will remain important for minimising EGM accessibility among vulnerable communities. Public involvement in EGM policy decisions has strong implications for policy effectiveness. Given the expanding range of gambling activities (including online gambling) presently accessible to communities worldwide, both organisational and public policies (as advocated through the programme) are needed to minimise gambling harms.

## Background

In the past few decades, types of gambling products and availability have increased worldwide, including high-risk products such as electronic gaming machines [1, 2]. Growing recognition of the broad range of harms that can occur with excessive gambling behaviour (e.g. relationship disruption, health decline, productivity decrease) and how these affect not only gamblers but their families and wider communities [3, 4] is shifting the focus from a treatment alone approach to one that includes harm minimisation and prevention. Prevention measures have included recommendations for, and enactment of, a range of policies in different jurisdictions. These include policies restricting who can gamble, mandating responsible gambling, regulating gambling profits redistribution, limiting gambling availability, and restricting gambling advertising [5–10]. New Zealand’s *Gambling Act 2003* goes further in mandating an integrated problem gambling strategy that includes public health programmes to prevent and minimise gambling harms [11]. The New Zealand Ministry of Health is responsible for implementing this strategy. Following development phases (2004–2009) [12–14], five national-level public health programmes were defined in the Ministry’s 2010 strategy [15], which focused on gambling-related policies, safer gambling environments, problem gambling screening, community awareness and community resilience. These programmes are implemented by public health service providers (implementers) located throughout New Zealand. These include small implementers (with few staff) covering small townships or rural areas, and two large implementers with offices in multiple locations. As a majority of implementers are also contracted to deliver treatment services, there is often collaborative work between counsellors and public health staff in delivering the public health programmes.

This article focuses on one of these programmes—Policy Development and Implementation (PDI). The programme’s overarching objective is to increase adoption of harm-reduction policies at organisational and local council levels [16]. To our knowledge, this is a unique and unprecedented approach to gambling harm reduction. Although policy development, policy change advocacy, and efforts to enhance law enforcement are among preventive programmes described in Mitchell et al.’s [17] typology of prevention activities for substance abuse, policy development has rarely been broached in the context of a public health approach for reducing gambling harm.

The PDI programme has three policy focus areas. The first, on *workplace/organisational gambling policies*, aims to increase voluntary adoption of policies that support gambling harm reduction among employees and/or clients of different organisations [16]. Several authors have discussed the need for *workplace gambling policies* (that address employee gambling while at work) and some have suggested policy content [18–23]; however, there is little evidence of real-life policy examples. Paul and Townsend [22] noted that part of the dilemma for employers in dealing with gambling in the workplace was that it required a working knowledge of relevant laws, i.e. of laws that apply to other workplace problems that may sometimes apply to the problem of employee gambling while at work.

Likewise, there is minimal evidence of *organisational gambling policies* on dealing with clients or service users who may be experiencing gambling harms. The limited evidence largely focuses on educational institutions that highlight how gambling policies are not as prevalent as, for instance, student alcohol use policies [24–27]. Where they existed in other settings (e.g. in some senior residential centres), they were informal practice (e.g. limiting trips to gambling venues), rather than formally written policies [28].

The PDI programme’s second focus area on voluntary *non-gambling fundraising policies* aims to encourage “methods of fund-raising that do not involve gambling” and influence organisational “positions on accepting gambling funding” (p. 30) [16]. However, gambling-based fundraising activities, i.e. games of chance such as bingo, raffles, lucky dips and lotteries carried out to generate revenue, have long been a common practice among community and social groups in New Zealand. Applying for gambling-proceeds grants is also common. Regulated by the Department of Internal Affairs, gambling proceeds from electronic gaming machines (EGMs) are distributed by EGM societies^1^ to community groups in the form of grants [29]. In 2015, societies were mandated to return a minimum of 40% gross proceeds (excluding goods and services tax) to communities [30]. While total returned proceeds vary from year to year, in general, these amount to over NZ$300 million per annum [31].

While policies governing the conduct of charitable gambling activities [32] and policies mandating the redistribution of gambling proceeds to public good sectors such as education, healthcare, and social services [9, 33] exist in some jurisdictions, policies mandating non-acceptance of funds from gambling proceeds or non-engagement in gambling-based fundraising activities are not common. Among hospitals in Ontario, Canada, that practice gambling-based fundraising, there was little consideration of risks and impacts and only a very small percentage had policies to guide such practice [34]. This policy area has a strong ethical basis; as Adams [35] argues, those who accept gambling funding indirectly endorse gambling participation and convey a false message that participation contributes to positive outcomes.

While gambling policies such as those above are confined within organisations that choose to adopt and implement them, public policies at a local jurisdictional level have a wider outreach as they target entire populations. The PDI programme’s third focus area on *council policies on EGM venues* aims to encourage the inclusion of community concerns about EGM venue density and locality in local council policy planning and implementation [16]. This policy is especially important as EGMs are strongly associated with problem gambling in New Zealand and elsewhere [36–38]. In New Zealand, all local councils are mandated by the *Gambling Act 2003* to adopt policies on EGM venues^2^ taking into account social impacts on deprived communities within their respective districts [11]. These policies guide the licencing of venues within councils. Council decisions on the planning and implementation of these policies are dependent on public response (e.g. public submissions) and evidence of social impacts in respective districts. One policy decision example is colloquially referred to as a “sinking lid” policy approach—following closure of an EGM venue, consent is not provided by the council for its relocation or replacement, thus reducing EGM venue numbers (and the potential associated harm) within the region [39].

To encourage the development of the abovementioned policies, the *service specification* for the PDI programme requires that implementers identify appropriate stakeholder organisations (e.g. councils, businesses, education providers, sports clubs, and community organisations) to work with, and develop relationships with; educate organisations on gambling harms and enhance their understanding of policy relevance; and facilitate community action [16].

Given the novelty of this ongoing public health programme, an evaluation to generate evidence about its implementation and progress appeared important. Unlike clinical interventions (that can be assessed using randomised controlled trials) and standardised programmes (that can be assessed using representative national surveys), public health and community-based programmes implemented in public settings are complex, multifaceted, and organisationally elaborate—placing them beyond the scope of positivist research models [40–42]. In our evaluation planning, an additional layer of complexity was brought about by the PDI programme’s nationwide delivery by 17 implementers in variable settings (i.e. cities, small towns). A multi-site evaluation taking into account local community and stakeholder perspectives was beyond our scope of inquiry. Furthermore, the evaluation took place while the programme was already in implementation ruling out a before-after evaluation design.

To offer implementers practically relevant information for programme improvement, we carried out a process evaluation to gain insights into programme implementation [43]. Rather than an assessment of a programme’s overall merit or success (as provided in an outcomes evaluation), a process evaluation aims to gather information (e.g. programme fidelity; programme strengths and limitations in terms of structure or processes; programme reception; and programme settings or contexts) that can explain programme outcomes (or lack thereof) and be used by programme planners and implementers to reinforce programme effectiveness [41, 43, 44]. A process evaluation thus enables identification of programme features that might influence programme success.

Here, we report insights from our evaluation based on 3 years’ of data (July 2010 to June 2013) following implementation of the PDI programme. Combining the experiences of different implementers, we report systematic processes, challenges to be anticipated and possible ameliorating actions that can inform similar public health programmes elsewhere.

## Methods

### Evaluation design

As the goal of our process evaluation included determining fidelity in the PDI programme’s implementation (i.e. the extent to which implementers followed recommended processes and delivered expected activities), we developed evaluation criteria (see Table 1) using the Ministry’s official *service specification* for this programme [16]. These criteria guided our evaluation questions, analysis and reporting. For instance, the programme’s *service specification* prescribed a process that included “identification of relevant organisations” and “relationship building” (p. 30) [16]. Therefore, “target group identification” and “relationship development” were among our evaluation criteria, and our evaluation questions included the extent to which implementers identified appropriate organisations for the different programme components, and how relationships were developed with these organisations.

**Table 1:** Evaluation criteria

Evaluation criteria	What was measured and analysed
Target group identification	• Range of target organisations/groups identified for the different programme components
	• Range of partner groups identified for collaborations
Relationship development	• Methods used to attract participation of individuals, groups and organisations
	• Relationship development processes and methods
Resource development	• Types and features of resources developed
	• Use of resources and resultant effects
Awareness raising	• Types and features of awareness activities delivered
	• Advise on gambling harm significance provided to organisations
	• Policy relevance communicated to organisations
	• Target organisations’ receptions/reactions
Policy development	• Types and range of policy lobbying and advocacy activities delivered and target organisations’ reactions
	• Types and range of policy development and implementation support provided to organisations
	• Methods used to encourage community involvement and action
	• Achievements in the three intended policy focus areas
Context	• Barriers, challenges and external factors that could potentially influence programme implementation and/or outcome

### Data and analysis

Our primary data set was over 100 six-monthly progress reports (official records of program implementation) that the 17 contracted implementers submitted to the Ministry between 2010 and 2013. These reports detailed required inputs, planned activities, outputs, outcomes (anecdotal descriptive accounts based on implementers’ observations or informal evaluation findings), challenges faced, and ameliorating actions. We noted some limitations in this data set including varying report formats, and lack of details such as participant numbers, explicit connections to the Ministry’s *service specifications*, and the prescribed outcome indicator (number of organisations adopting advocated policies). Understandably, implementers’ workload may mean less than complete or up-to-date reporting on programme delivery and consequently diminished quantity and quality of data available to evaluators [45].

We carried out a *document analysis* of the progress reports and employed an integration of *thematic* and *content analysis* as recommended by Bowen [46]. To clarify some findings from our *document analysis*, we sought the perspectives of eight public health staff from six implementer organisations (selected to represent urban and rural locations and ethnic-specific services) in a focus group discussion. The focus group was digitally recorded and transcribed. Themes in both progress reports and focus group transcripts were largely identified in a deductive manner (theoretical thematic analysis), as the evaluation was concerned with identifying aspects of the data that fit with a pre-determined coding frame [47] based on the evaluation criteria described in Table 1.

Using a logic model framework of programme delivery [48–50], in our analysis, we identified the inputs, outputs, and outcomes that implementers reported. As each implementer’s report spanned 3 years, we were able to track progress of programme components over time—from a planning and preparatory phase to delivery and outcomes. Additionally, an inductive thematic analysis enabled identification of additional and unexpected aspects in programme delivery and external factors (beyond the control of implementers). External factors are particularly important to consider for programmes delivered within the public arena as these factors, potentially, can negatively or positively influence outcomes [49]. The *content analysis* method that enabled an estimate of theme frequency across the progress report data set (i.e. representing *all*, *most*, *some*, or *a few* implementers) was to provide a national overview of strengths and challenges in program implementation. Key themes with supporting extracts from implementer reports and focus group transcripts were verified by members of the research team.

## Results

### Awareness raising and relationship development

*Awareness raising* was a key output-related theme noted in our document analysis for all three policy focus areas for all implementers. This confirmed process fidelity, as raising awareness were among activities prescribed in the *service specification*. Most implementers delivered purposeful awareness-raising activities to enhance understanding about problem gambling, gambling harms and the need for the respective policies they were advocating. Some implementers’ informal evaluations suggested these activities resulted in positive outcomes—enhanced knowledge, affected perceptions, and increased willingness to participate in policy development.

*Relationship development* with stakeholders (as prescribed in the programme’s *service specification*) was another overarching output-related theme noted in all implementer reports for all three policy focus areas. Although implementers targeted and worked with different stakeholder organisations for the three policy focus areas, the importance of relationship development was universal and resulted in several advantages. Stakeholder relationships were important for programme success, as implementers were able to identify allies and collaboration opportunities.

*Our relationships with other organisations and networks have demonstrated that we have numerous allies in the goal of reducing the harm caused by problem gambling.…Because of the relationship [we have] developed with key…agencies…we were invited to present information about gambling and gambling harm…at [a] health advisory group meeting.*

Some highlighted the value of strategically seeking influential stakeholder organisations, particularly those with connections with Māori^3^ and Pacific communities—two groups that are at substantially higher risk of developing problem gambling in New Zealand [36]. Relationships with appropriate stakeholder organisations connected implementers to communities and enabled a wider audience reach and inclusive forums for policy discussions.

Implementers also stressed the importance of relationship maintenance through on-going visits to stakeholder organisations and described successful relationship development as a transition from an awareness-raising phase to a mutually beneficial working relationship phase.

Stakeholder relationship development was however time consuming. Some implementers found themselves being passed from one person to another when dealing with stakeholders that lacked clear processes for considering new workplace/organisational policies. Others reported bureaucratic processes within public health services, which deterred collaboration. Implementers’ resource limitations were compounded when they covered more populated territories or larger geographical areas, or needed to convey awareness messages in multiple languages. Collaborations between implementers provided a partial solution to resource limitations. One implementer believed such collaboration to be advantageous as it displayed a ‘unified front’ in terms of a commonly shared goal for gambling policies.

### Workplace/organisational gambling polices

**Fig. 1:**
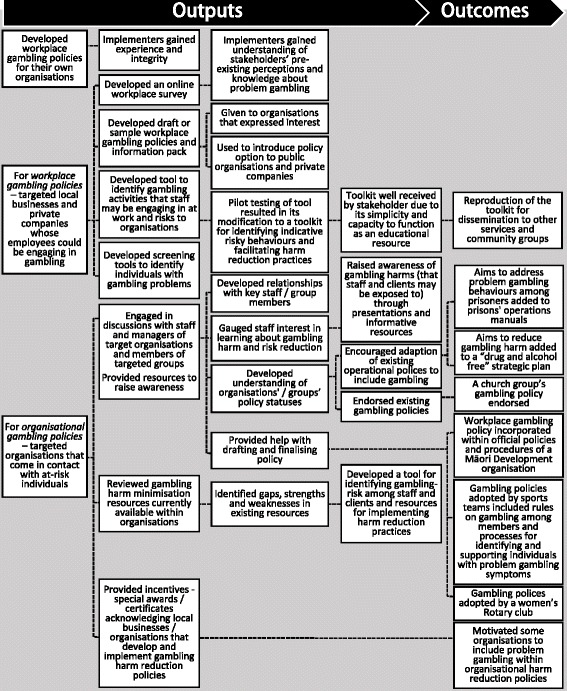
Processes leading to workplace/organisational gambling policy development

The processes and outputs and outcomes that implementers reported in efforts to encourage *workplace/organisational gambling policy* development are summarised in Fig. 1.

For *workplace policies*, local businesses and private companies (whose employees could be engaging in gambling) were among stakeholder organisations targeted by some implementers. Experiential knowledge may be considered an essential additional input for this programme component as a few implementers highlighted the need to first develop their own workplace gambling policies. This equipped them with the experience and integrity to provide policy advice to other organisations. Implementers also developed tools and resources, and sample policies, to aid their advocacy work.

*Our…team has been leading the development of a workplace policy. A first draft of the policy has been written. Next steps include peer review, and then introduction into workplaces around the…region.*

Implementers then initiated dialogue with target stakeholder organisations to gauge their interest, pre-existing policies, and resource needs. This knowledge helped them decide on appropriate awareness materials and advocacy strategies to encourage inclusion of problem gambling in workplace policies. Awareness raising efforts included presentations to company managers. Another strategy used was workshops delivered to businesses to facilitate an understanding of how gambling harm reduction policies might benefit the organisation; for instance, by offering a preventive measure against misuse of company finances or other resources.

*The focus…[was] to increase understanding of the necessity for policies which minimise harm from gambling as a measure to protect the organisation and its staff from developing unhealthy gambling habits with subsequent aversion of the temptation to take unfair advantage where contact with money or access to finances are a part of the core business.*

A few implementers offered these organisations’ tools to identify gambling in the workplace and assess financial risks, and informational resources to enable those requiring support to access specialist assistance. In a few cases, implementers also offered simple incentives (e.g. special awards, certificates) that recognised development and implementation of policies. A few implementers took a more direct approach of actively supporting organisations in drafting and finalising policies.

As shown in Fig. 1, for *organisational policies*, stakeholder organisations that implementers targeted were social services, corrections facilities, sports groups, banks, prisons, mental health services, suicide prevention programmes, alcohol and drug treatment services, and community groups (all likely to come in contact with individuals who may be experiencing gambling harms) and local services such as libraries that provide internet access. In a few cases, specific departments within these organisations likely to be responsible for health-related policies (e.g. Human Resources, Health and Safety) were targeted. With these organisations, the dialogue focus was on highlighting problem gambling as a possible cause of social, health, and financial issues. These organisations were also offered tools to identify problem gambling among their clients/service users and resources to enable implementation of harm reduction practices.

As shown in Fig. 1, implementers’ efforts led to the establishment of some workplace/organisational gambling policies*.* However, they also identified several challenges. Getting organisations to recognise problem gambling as a relevant issue was often an initial hurdle. Resistance to change due to pre-existing perceptions was also common; for example, a view among these organisations’ personnel that they were not responsible for gambling harm reduction policies; that gambling was a personal rather than a “public safety” or “health” issue; that gambling harm was not prevalent or a workplace issue; and that existing health-related policies were adequate.

*…the common theme among managers is that gambling couldn’t cause issues for the organisation, thus being, there was no need to consider gambling within their policies.*

*Organisations believe they have adequate policies in place and are not necessarily willing to engage in extra policy development.... The academic fraternity, bigger NGOs and health service providers generally do not have specific problem gambling harm-minimisation [policies], [but] some feel their health and safety [policies] covers this type of harm.*

These challenges were addressed through provision of additional information. For example, beyond highlighting the prevalence of problem gambling, implementers found that it was important to ensure that organisations developed an understanding of problem gambling symptoms, resultant financial, social and health effects, and other “hidden” gambling harms.

The lack of any legal requirement to adopt gambling-related policies was also a disincentive.

*Organisations mainly seem interested in developing and implementing gambling [policies] only if it is a legal requirement or compliance issue [regarding] employee initiatives*.

Additionally, when there were other pressing issues (e.g. structural changes) or work priorities, or time or resource limitations, stakeholder organisations were less enthusiastic about adopting workplace or organisational gambling policies. Perceptions that policy adoption would result in financial or administrative costs were also impediments. To overcome these barriers, implementers described the importance of understanding the contexts of these organisations before deciding strategies to influence policy uptake. For instance, conveying policy benefits, and deflecting perceptions that policy uptake would increase workload or incur substantial costs.

*It has been important to understand the work practice of organisations, so as to shape policies, which enhance rather than give the perception of ‘more work’.*

Implementers also reported that problem gambling screening tools offered to organisations should be simple (requiring minimal time) and that the availability of free support from problem gambling clinical services should be highlighted to reverse misconceptions about additional costs to organisations.

Some implementers found that community groups with members who were social gamblers were resistant to organisational gambling policies. Perceptions of gambling as a social activity and a readily available entertainment also posed a challenge, particularly in lower socio-economic communities. For such cases, implementers highlighted the importance of understanding community contexts, for instance, the locations of gambling venues in the area, in addition to enhancing awareness of the broader nature of gambling harms, including financial losses and impacts on communities and families.

A few implementers who were unsuccessful in encouraging policy development at the time of their reporting believed that having established working relationships with these organisations was an important first step towards future success. Because of the PDI programme, some organisations acknowledged the reality of gambling harms and had better awareness of available support services.

*Development of harm-minimisation policies for the majority are not a priority at this stage. However, gambling harm is now widely acknowledged and known in the workplace and organisations are now more aware of who to contact and refer to when gambling harm arises.*

### Policies on non-gambling fundraising

Implementers’ advocacy for *policies on non-gambling fundraising* were targeted at stakeholders such as community groups, sports clubs and church groups that typically rely on gambling-proceeds grants and engage in gambling-based fundraising activities (e.g. raffles). Their efforts included raising awareness of gambling harms and facilitating discussions on alternative fundraising methods with these groups. One implementer reported successful outcomes following their awareness raising work with a community organisation, which led to the organisation’s formulation of a non-gambling fundraising policy.

*For some time, [we] worked with a branch of the Māori Women’s Welfare League to raise awareness of gambling harm. We are pleased to report that the League has recognised the issue of gambling harm and formulated a policy to guide its members. The League has taken the position that they will not apply for …casino gambling proceeds to support their activities given the harm caused by gambling.*

However, this policy development was not widespread. Other implementers’ reports suggested that efforts remained on encouraging attitude shifts among target groups by discussing alternative fundraising methods (e.g. food sales, garage sales). Several implementers reported difficulty in identifying and securing alternative funding sources for groups that had become reliant on funds from gambling proceeds.

*Community providers have acknowledged gambling harm and the potential difficulties associated with fundraising activities which are gambling based. However, providers have also struggled to identify other ways in which they can raise funds.*

In our focus group discussion, we mentioned these challenges and queried if there had been additional efforts to enhance programme effectiveness. The discussion confirmed no further progress beyond encouraging alternative fundraising practices such as sales of traditional food, hand-made culturally meaningful products and tickets to cultural performances—activities that also strengthened cultural identity. Focus group participants also expressed views that this policy’s focus (i.e. non-acceptance of gambling funding) was somewhat controversial. Their observations were that groups that applied for and benefited from gambling-proceeds grants were often from higher socio-economic communities rather than from the poorer communities experiencing gambling harms. They believed that dissuading poorer communities from applying for these grants could exacerbate this unjust transfer of gambling proceeds.

### Council policies on EGM venues

**Fig. 2:**
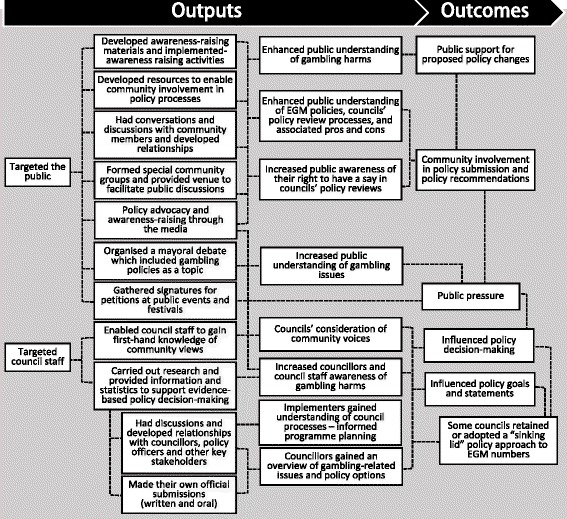
Council EGM policy lobbying processes

The outputs and outcomes implementers reported in their efforts to influence council *EGM policy* decisions are summarised in Fig. 2. They targeted two main stakeholder groups—the general public and council staff. One implementer also targeted local businesses that could be experiencing indirect effects of gambling; for instance, rental property companies with tenants unable to pay rent may consider problem gambling as an underlying cause and be likely to support EGM venue policies.

Essential inputs that many implementers reported included awareness-raising materials such as policy factsheets and posters with information on council policy review processes; guides to making submissions; and materials to enable community participation (e.g. submission templates, petition forms). The materials helped individuals to personalise their submissions and as explained by an implementer, the materials were necessary “to make it easier for the community to have their say” in the policy process. Materials were delivered directly to target groups and placed in public facilities (e.g. libraries, community centres). Community members were encouraged to comment on the EGM numbers in their region and resultant effects on their community. Awareness raising was also carried out in education booths at public events. Some implementers gathered signatures for petitions at public events and festivals to support their policy advocacy work; formed special groups and organised community meetings to build understanding of the policy submission process; facilitated workshops on preparing for oral submissions; and provided venues for people to discuss gambling harms.

*[We] began hosting community [meetings to explain] the Council processes…[and provided] resources to assist them in completing a submission. Feedback received has been good with the majority of the people in attendance [indicating they were previously unaware] that they were able to have a say in local council policies.*

*[We]…initiated an action group with five participants …This was an energised group who were eager to inform professionals and the general public about the Class 4 gambling review. The group supported people to make submission[s] and encourage a “sinking lid” submission …*

A few implementers encouraged involvement from current and former users of problem gambling treatment services, as insights from those individuals were regarded as important for informing policy decisions.

*[We] advocated for a separate oral hearing for our problem gambling clients. [They]…were allowed to talk in private away from media and pokie* 4 *industry as they were telling their stories of harm and recovery to the … Council hearings committee. …At the time of writing this report the hearings panel has recommended a “sinking lid” policy.*

Awareness was also promoted through some implementers’ websites; social media channels such as Facebook and Twitter; and radio, print, and television media. Following interviews in a live phone-in radio programme, an implementer reported receiving numerous calls from the public requesting information and policy submission forms. Press releases, letters to the editor, media interviews, and advertisements were used to spotlight the importance of public involvement in EGM policy reviews. Media strategies used by a few implementers included inviting press coverage of oral submissions, and participation in demonstrations that attracted media attention.

As listed in Fig. 2, a few implementers enhanced community involvement by enabling council staff responsible for policy development to gain first-hand knowledge by directly hearing community voices. Council staff were invited to attend community meetings; councils were offered support to organise community consultations; and arrangements were made for community members to speak at official hearings.

*[We] had the honor of escorting a group of respected Māori elders to the Sinking Lid review, where many of them stood and spoke of the effects [of]gambling…which carried a heartfelt message to the reviewing panel, and the outcome of the review was a positive result, with the sinking lid being maintained.*

To raise awareness and build relationships, a few implementers also participated in meetings and forums where they discussed issues with city councillors, policy analysts, and other key stakeholders responsible for policy decision-making. These conversations were geared towards answering the “why should we?” question in terms of EGM policies, and directing attention to connections between problem gambling and other social problems (e.g. domestic violence, crime) and the importance of public involvement. Relationships with council staff led to invitations to carry out informational workshops. Implementers’ interactions with council staff gave them a better understanding of how councils functioned and, in turn, this enabled their programme planning.

*…our public health workers receive positive feedback …from their local councils …regarding the information and work provided to support gambling policy development. …open and collaborative relationships with council staff [lead] to invites around workshop presentations and information gathering…*

Most implementers made substantial preparations to support councils’ evidence-based policy decisions. Their preparations included reviewing existing EGM policies and long-term council plans to identify gaps (e.g. social development or community wellbeing components) which they then used in a policy lobbying process during discussions with the respective councils. Their policy lobbying was supported by gambling-related statistics, facts on gambling harm, insights from problem gambling treatment services, and relevant socio-economic data from community support services. To encourage recognition of the importance of the “sinking-lid” policy approach within a council, one implementer highlighted the commitments of other councils that were supportive of this approach as reflective of “innovative community leadership”. Most implementers also made their own official submissions (written and oral) to respective councils in the period preceding an EGM venue policy review.

*Statistics from the local food bank examining poverty levels were also obtained for the oral submission to the Council… [Our]…submissions contained generic information about gambling and gambling harm and included local statistics and information about gambling harm specific to the Local Board area.*

*[Our presentation to the Council’s annual plan hearing which contained]… relevant demographic facts and statistics … was well received. Most of the councilors were supportive of a “sinking lid” policy and were amazed by the statistics.*

Outcomes included increased public awareness about gambling harm and of their right to be involved in their council’s policy review. Community involvement in the policy process generated public pressure (either directly through submissions or indirectly through the media), which some implementers believed influenced policy decisions. As shown in the outcomes column in Fig. 2, successful influences were largely related to councils’ decisions to adopt or retain the “sinking-lid” approach, which reduced EGM venue numbers within a district through council-imposed restrictions.

However, some implementers reported that the policy decisions of some councils remained unaffected despite substantial implementer lobbying and community involvement. When voting, more council members rejected the “sinking-lid” approach or weakened the approach by introducing a clause allowing venues to relocate. For these councils, ongoing awareness building and lobbying were identified as necessary.

Perceptions among council staff of the value of gambling revenue for boosting local economic development and funding of community groups were a common barrier.

*[We] participated in a forum where representatives from community boards and councils attended to voice their views on the policy and whether a sinking lid policy should be implemented. …only a few support[ed] a sinking lid policy, the remaining were of the opinion that the community wasn’t largely affected by gambling and was well supported by the funds from the Trusts [i.e. EGM societies].*

*[The] Council declined implementing a sinking lid policy, stating that the economic benefits from these machines would help boost the district and a sinking lid policy will disadvantage potential business developments in the area.*

A few implementers reported that when city councillors seemed to have vested interests in the EGM business, this barrier was amplified. Other challenges included lack of clarity in the timing of councils’ EGM policy reviews and uncertainties following structural changes (e.g. not knowing the decision-making priorities of new councillors). One implementer reported that it was often difficult to know who among a new council would be supportive of their PDI programme aims, and if a new council rejected a recommended policy change, then additional community rallying was required. Another implementer’s process of sending self-introduction letters (explaining the programme they offered within the community) to all new councillors offered a way to address this hurdle by opening up communication channels.

One implementer’s view was that a council’s prioritisation of gambling policies could be influenced by various factors: (1) perceptions among council staff that problem gambling was not prevalent in their community or that it was a central government issue; (2) councillors’ workload and competing demands on their time and resources which shifts their priorities elsewhere; (3) costly public consultation processes required for policy amendment acting as a deterrent and steering councils to roll over existing policies instead; and (4) lack of robust processes for collecting evidence on the social impacts of gambling harms to support advocated changes during a policy review. This implementer believed available social impact data was often anecdotal and subjective.

Resistance and pressure from gambling venue operators was an external factor with the potential to influence council decisions. For instance, one implementer reported that in a council hearing, those opposing the sinking-lid policy approach were mainly EGM proprietors or representatives of EGM societies.

## Discussion

In this article, we report insights from a first-time process evaluation of a nationally implemented public health programme on gambling policy development and implementation (PDI), comprising three policy focus areas. Our findings on implementers’ outputs, experiences (challenges and ameliorating strategies), and successful cases of policy lobbying offer a useful compilation of processes that may serve to guide similar policy-focused public health initiatives in jurisdictions experiencing growth in gambling activities, particularly, the high potency continuous types, and corresponding increases in gambling harm [1, 51].

Implementers reported developing tools, resources and awareness-raising materials to aid their policy advocacy work. The ability to develop resources may be regarded an essential input area for programme success. Efficiency of this input area could be enhanced through a formal process for national-level resource sharing.

Findings showed the PDI programme resulted in development of some workplace/organisational gambling policies, little development of policies on non-gambling fundraising, and some positive influences on councils’ decisions on *EGM policies*. The range, complexity, and enormity of challenges faced by implementers in advocating these policies were unsurprising considering the novelty of gambling policy development. As Gainsbury and colleagues [52] noted, unlike availability of evidence-based guidelines for developing public policies on alcohol, tobacco, and illegal substances, “no corresponding standards have been developed to guide regulators in establishing evidence-based gambling policies” (p. 773). Workplace/organisational gambling policies and non-gambling fundraising policies remain avant-garde policy concepts, unheard of in most organisations, and have little policy development guidelines.

Implementers faced challenges in all stages of their policy advocacy process. Prior to recognition of policy, there needs to be problem recognition and definition [53]. Our findings highlighted the importance of addressing the ambivalence concerning the seriousness of gambling issues for gambling policy development. Implementers’ experiences suggested that for organisations to consider workplace/organisational gambling policies, they first need to be convinced that problem gambling was a public health issue (not merely a personal issue). They also noted that appreciation of the wider public health value of policies that address gambling harms among an organisation’s clientele was a prerequisite for *organisational gambling policies*. The “problem” thus needed to appear large to justify organisational action. This required extensive awareness building on the nature of problem gambling and its prevalence on the part of implementers and may be aided by wider national-level awareness raising of problem gambling as a public health issue. Essential attitudinal changes are likely to occur following prolonged advocacy efforts by public health programme implementers and formation of collaborative working relationships with stakeholder organisations. For community groups that tend to see gambling as a social activity or entertainment, in addition to evidences of gambling harms, availability of alternative social activities could encourage their consideration of gambling policies. Other challenges implementers encountered in advocating workplace gambling policies were similar to those described in the literature. A reluctance to acknowledge workplace gambling; concerns over the implications that such an acknowledgement would instigate; preference to deal with gambling problems on an ad-hoc basis; uncertainty about legal obligations; and concerns over potential costs may prevent the inclusion of gambling in workplace policies [18, 19].

Implementers’ experiences suggested that prerequisites for workplace policy uptake include evidence of the range of risks and costs to employers that can result from gambling in the workplace. This could be made a critical component of the PDI programme’s contents as suggestions for advocating workplace gambling policies in the literature similarly mentioned evidences of costs and risks to employers in the form of absenteeism, weakened productivity, effects on co-workers, theft, and embezzlement [18, 19, 21, 54–56]. Considering the ease of undetected Internet gambling at work, Griffith’s [19] proposal for workplace gambling policies emphasised the need to recognise such gambling as a form of employee Internet abuse. It appeared that in addition to enhanced understanding, changes in attitude are required before such policies are seen as important for workplace wellbeing, and gambling harm reduction is considered to be similar to workplace health and safety concepts such smoke-free, drug-free, and safe drinking [57, 58].

Advocating policies on non-gambling fundraising was the most challenging of the PDI programme components. For the majority of implementers, progress was restricted to encouraging alternative fundraising activities. Implementers highlighted the heavy reliance of some community groups, schools, and sports teams on grants from gambling proceeds as a barrier. Understandably, without comparable alternative funding sources, adopting a non-gambling fundraising policy would make little sense to these groups. In the face of diminishing government contributions, community groups and public good organisations are drawn to the more easily accessible gambling-proceeds grants and subsequently develop a dependence on them [59].

Overall, implementers’ experiences and views implied that the programme component focused on non-gambling fundraising policies was at odds with regulations governing the distribution of gambling revenue via gambling-proceeds grants. EGM societies are bound by a mandated minimum rate of return that specifies the percentage of gross proceeds (e.g. 40% in 2016) to be returned to communities each financial year [30]. This reflects the reality of a government’s position, which entails involvement in all aspects of gambling—taxing, policing, and regulating industry while funding help services for those experiencing harm from gambling [60].

The transfer of financial resources from poor to rich occurring through distribution of grants from gambling proceeds, commented on by implementers in our evaluation, was comparable to observations of racial inequity creation in Australia through transfer of gambling profits from disadvantaged remote areas (resided by Aboriginal communities) to sites of predominantly white communities [61]. Other areas of concern in such community contribution systems may also include lack of transparency in self-reporting by the EGM industry as has been identified for the Australian Capital Territory [62]. In New Zealand, although there are currently no mandatory restrictions on the geographic distribution of gambling proceeds, many EGM societies have adopted localised returns policies for channelling proceeds back to the community from which they were generated [31]. It may be beneficial for PDI programme implementers to work collaboratively with EGM societies and to reconsider the target sector for non-gambling fundraising policies development. For instance, steering focus to groups from higher socio economic areas or resource-rich groups with greater capacity for seeking and organising alternative funding. Additionally, gambling-proceeds grant recipients could be encouraged to promote gambling-harm minimisation messages in their activities.

The ethical and moral implications of charitable funds [59] are worthy of deliberation as ethical and moral concerns are often determining factors for non-acceptance of funds from gambling proceeds. Of 647 registered charities in Canada, 63% that did not use gambling-based fundraising methods indicated ethical concerns as a deciding factor [63]. Advocates of non-gambling fundraising policies may also benefit from considering a method used in Alberta, Canada, for assessing willingness to pay (among those guided by moral norms about gambling) for public good activities such amateur sports and recreation programmes via income taxes [64]. However, although an ethical stance is helpful, dependence on funding from gambling proceeds may outweigh moral norms when groups believe they have no other choice [63]. Therefore, encouragement of non-gambling fundraising policies should coincide with providing groups with alternative funding sources and fundraising ideas. The framing of gambling and related social issues can affect public policy debates by either facilitating such debates or curtailing them [65]. Therefore, non-gambling fundraising policies may also need to be supplemented with enabling policies controlling the framing of gambling-based sponsorships; for instance, what Harris [66] argues to be promotion of a “false altruism” by gambling industry public relation professionals who highlight the benevolence and charitableness of the industry while negating the true nature of these funds (i.e. that it includes gambling losses from vulnerable individuals). It appeared that the PDI programme component on non-gambling fundraising policies requires additional measures (e.g. debates on the ethics of charitable funds) and other enabling corresponding policies (e.g. control over charitable funds rhetoric), without which, any immediate outcomes in the form of policy development are likely to remain within small circles of community groups.

Through the PDI programme, implementers successfully influenced some councils’ decisions on retaining or adopting the “sinking-lid” approach to *EGM policies* (which aims to reduce EGM venue numbers within a district through council-imposed restrictions). These outcomes required extensive time and resources, as aiding councils’ informed decision-making and public involvement were key features. The latter feature has potential to empower communities by enabling their inputs into policy decisions that can affect them, which in turn, has strong implications for policy effectiveness [67, 68]. Success was dependent on overcoming challenges such as perceptions among city councillors about the economic benefits from gambling revenue, and of problem gambling as a relatively minor issue in their community. Various strategies proved effective, including strategic communication in the advocacy process (e.g. highlighting best practices from other districts; connecting problem gambling with high priority social issues such as domestic violence and crime) and evidence-based lobbying (e.g. provision of facts and statistics on gambling and gambling harms). The latter may be regarded an impactful approach as “evidence” is a key determinant of public health policy [69, 70]. The strategy of enabling the participation of those directly affected by gambling harm in the policy lobbying process noted in our evaluation may also be seen as a potentially impactful approach as the “special political influence” they have are among determinants of public health policy [69]. Additionally, general public participation is also important, as gambling policy adoption (or lack thereof) and the shaping of gambling policies tend to be dependent on the nature of general public opinion (positive or negative) about gambling [71, 72].

While the PDI programme demonstrated some success in achieving intended policy influences, future evaluations will need to consider EGM policy impacts on gambling behaviours. EGM expenditure trends between 2007 and 2012 indicate that council policies did not result in substantial or uniform effects in terms of reducing player losses [73]. This may be due to people increasing use of other nearby venues following closure of local venues, or gambling the same amount on fewer machines [73]. Furthermore, the sinking-lid policy approach is mitigated by the fact that it only comes into effect six months following licence cancellation or surrender—a timeframe within which new licences may be applied for [73]. Even so, by influencing council EGM policies in areas populated by vulnerable communities, the PDI programme offers a targeted harm reduction approach. This policy focus area has the potential to reduce the higher concentration of EGM venues in lower socioeconomic regions observed in New Zealand, and may be beneficial if also implemented in other countries such as Australia where similar distributions of EGMs are evident [74–76]. The links between gambling availability and higher levels of gambling engagement and problem gambling prevalence [77–79] also amplifies the importance of this policy focus area.

To summarise, in this paper, we focused on the process aspects of a public health programme on gambling policy development and we highlighted some key policy development approaches and policy determinants necessary for successful outcomes. Due to the programme’s delivery circumstances, ours was a process rather than an outcome evaluation. Impacts of actual policies developed and implemented as result of this programme were beyond our evaluation scope, limiting our capacity to discuss the harm reduction benefits of the programme. Considering other limitations of our evaluation (reliance on variable implementer progress reports as the primary data source) and the context-dependence of public health programmes, we do not claim generalisability of our findings. A long-term outcomes’ evaluation that includes tracking of progress across time is necessary to determine the actual number of policies established via the programme. The harm prevention or harm reduction effects of the three policy focus areas of the programme would require empirical policy analysis that includes impact questions (both intended and unintended) [53]. Considering the high costs of a nationwide study, small-scale case studies on selected localities could be used to demonstrate policy impacts. Under enabling circumstances, a “before-policy, after-policy” outcomes’ evaluation may be a cost-effective method for assessing the extent of policy impacts [60].

## Conclusions

Both organisational and public policies are likely necessary to address negative impacts resulting from the wide range of gambling activities (including online gambling) presently accessible to communities worldwide. Our process evaluation of a pioneering national-level public health programme aimed at advocating gambling policies has enabled us to attest the potential of this public health approach for gambling harm reduction as the programme resulted in some policy development. Our findings on the processes that led to policy development and key policy determinants can both inspire and inform the development of similar public health initiatives elsewhere.

Workplace/organisational gambling policies could potentially encourage organisations to address the issue of gambling problems at a level comparable to other public health issues such as tobacco, alcohol, and drug abuse. In addition to awareness raising on the seriousness of gambling harms, evidence of the range of gambling-related risks and costs to employers appear essential for advocating these policies.

Policies on non-gambling fundraising, albeit challenging, have the potential to encourage creative and culturally significant fundraising practices that can enhance social cohesion (offering a social protective factor). Policy advocacy should include widening understanding of the ethical implications of gambling profits and provision of fundraising ideas and coincide with the availability of alternative funding sources.

*Council EGM policies* that impose restrictions on location and distribution of EGM venues, taking into account the socioeconomic status of local communities, can potentially curtail an over-representation of EGM venues within lower socioeconomic areas—offering precautionary steps for reducing harm among vulnerable communities with limited financial resources. Negating exaggerated perceptions of the economic benefits of gambling revenue; public involvement, including individuals directly affected by gambling harms in policy advocacy; and evidence-based lobbying were among key features for influencing EGM policies.

Overall, policy uptake was dependent on addressing the ambivalence concerning the seriousness of gambling issues and gambling policy necessity through awareness-raising activities, collaborative relationships, and an infused sense of a shared responsibility in addressing gambling harms. This paper is but a start to the debate on gambling policy development for harm reduction.

## Abbreviations

EGM: Electronic gaming machines
NGO: Non-Governmental Organisation
PDI: Policy Development and Implementation

## Footnotes

1 In New Zealand, EMG societies are licenced to operate gaming machines in commercial venues (other than in casinos).

2 These policies are referred to as Class 4 venue policies in the *Gambling Act 2003*; Class 4 venues are defined as those offering gaming machines outside a casino.

3 Māori are the indigenous people of New Zealand and currently comprise approximately 15% of the population.

4 Pokies (poker machines) is a common slang term used in reference to electronic gaming machines in New Zealand and Australia.
